# Insights into Biomechanical and Proteomic Characteristics of Small Diameter Vascular Grafts Utilizing the Human Umbilical Artery

**DOI:** 10.3390/biomedicines8080280

**Published:** 2020-08-10

**Authors:** Panagiotis Mallis, Dimitrios P. Sokolis, Manousos Makridakis, Jerome Zoidakis, Athanasios D. Velentzas, Michalis Katsimpoulas, Antonia Vlahou, Alkiviadis Kostakis, Catherine Stavropoulos-Giokas, Efstathios Michalopoulos

**Affiliations:** 1 Hellenic Cord Blood Bank, Biomedical Research Foundation Academy of Athens, 4 Soranou Ephessiou Street, 115 27 Athens, Greece; cstavrop@bioacademy.gr (C.S.-G.); smichal@bioacademy.gr (E.M.); 2 Laboratory of Biomechanics, Center for Experimental Surgery, Biomedical Research Foundation Academy of Athens, 4 Soranou Ephessiou Street, 115 27 Athens, Greece; dsokolis@bioacademy.gr; 3 Biotechnology division, Biomedical Research Foundation Academy of Athens, 4 Soranou Ephessiou Street, 115 27 Athens, Greece; mmakrid@bioacademy.gr (M.M.); izoidakis@bioacademy.gr (J.Z.); vlahoua@bioacademy.gr (A.V.); 4 Department of Biology, Section of Cell Biology and Biophysics, School of Science, National and Kapodistrian University of Athens, 161 Gr. Kousidi, Zografos, Street, 115 27 Athens, Greece; tveletz@biol.uoa.gr; 5 Center of Experimental Surgery and Translational Research, Biomedical Research Foundation Academy of Athens, 4 Soranou Ephessiou Street, 115 27 Athens, Greece; mkatsiboulas@bioacademy.gr (M.K.); akostakis@bioacademy.gr (A.K.)

**Keywords:** human umbilical arteries, decellularization, DNA, biomechanical analysis, LC/MS, collagen, fibronectin, cardiovascular disease

## Abstract

The gold standard vascular substitutes, used in cardiovascular surgery, are the Dacron or expanded polytetrafluoroethylene (ePTFE)-derived grafts. However, major adverse reactions accompany their use. For this purpose, decellularized human umbilical arteries (hUAs) may be proven as a significant source for the development of small diameter conduits. The aim of this study was the evaluation of a decellularization protocol in hUAs. To study the effect of the decellularization to the hUAs, histological analysis was performed. Then, native and decellularized hUAs were biochemically and biomechanically evaluated. Finally, broad proteomic analysis was applied. Histological analysis revealed the successful decellularization of the hUAs. Furthermore, a great amount of DNA was removed from the decellularized hUAs. Biomechanical analysis revealed statistically significant differences in longitudinal direction only in maximum stress (*p* < 0.013) and strain (*p* < 0.001). On the contrary, all parameters tested for circumferential direction exhibited significant differences (*p* < 0.05). Proteomic analysis showed the preservation of the extracellular matrix and cytoskeletal proteins in both groups. Proteomic data are available via ProteomeXchange with identifier PXD020187. The above results indicated that hUAs were efficiently decellularized. The tissue function properties of these conduits were well retained, making them ideal candidates for the development of small diameter vascular grafts.

## 1. Introduction

Modern cardiovascular surgery requires the use of suitable vascular grafts for bypass operations. Cardiovascular disease (CVD) is one of the leading causes of death worldwide [1]. CVD is a group of disorders, including peripheral arterial disease, coronary heart disease, cerebrovascular disease, and rheumatic heart disease. In most of these cases, bypass grafting with the use of small diameter vascular grafts, holds a promising solution. It is estimated that more than 17.9 million people are suffering from CVD. Additionally, each year more than 500,000 bypass surgeries are performed [2,3]. From an economical point of view, CVD costs annually more than 210 billion euros to the European Union (EU) and more than 300 billion dollars to the USA, indicating that better therapeutic approaches must be established [1,2,3,4]. Vascular applications, such as hemodialysis treatment and femoral popliteal bypass, also require vessel conduits for their performance. 

In cardiovascular reconstruction, the use of autologous vessels, such as saphenous vein, mammary and radial arteries may be used as a potential source of vascular grafts [5]. These vessels are fully compatible with the recipient, and are characterized by in vivo remodeling properties and antithrombogenic surface. However, only 40% of patients with CVD have suitable autologous vessels, which can be applied in cardiovascular surgeries [6]. Furthermore, when a second vascular replacement is required in the patient, the finding of a secondary suitable autologous vessel is quite a demanding task. In addition, the differences in mechanical properties between vessels (arteries and vein), can result in compliance mismatch, leading to aneurism formation, vessel occlusion, and rejection [7].

Currently, the gold standard conduits for CVD applications are the FDA approved vascular grafts derived either from Dacron or ePTFE [8]. Moreover, ePTFE based vascular grafts are used in the above and below the knee bypass surgeries, such as femoro-popliteal bypass. Indeed, these synthetic grafts have extensively been applied as substitutes for damaged large diameter vessels (d > 6 mm), maintaining approximately 80% of patency rate after 5-years of implantation [6,7,8]. In the case of small diameter vascular grafts replacement (d< 6 mm), the use of synthetic grafts is limited [9]. Significant adverse reactions such as Th1/Th2 response, intima hyperplasia (due to differences in mechanical properties), and thrombotic complications have been reported, decreasing significantly their implantation lifetime [8,9,10]. Moreover, the patency rate of ePTFE based vascular grafts, when applied as coronary artery substitutes, is below 60% within the first year of implantation and 32% within the second year. On the other hand, when the saphenous vein is used for the same vascular reconstruction, it is characterized by 95% and 90% patency rates within the first and second year of implantation, respectively [8,9]. In regards to below the knee bypass surgeries, ePTFE based vascular grafts also exhibited low patency rates (39% within the second year of implantation). To overcome these issues, a new surgical operation is required, in order to avoid life threating manifestations [8,9,10]. In the last decade, a great effort is performed by the research society to develop suitable vascular grafts including cellularized synthetic grafts, hybrid grafts, and natural polymer derived grafts, to improve further the patency rates. Additionally, the use of xenogeneic vascular grafts in cardiovascular operations, has been also proposed. However, animal-derived grafts are bearing a-gal epitopes, which can induce hypersensitivity reaction to the recipient, leading to calcification and graft failure [8,11]. 

In the context of vascular engineering, the human umbilical arteries (hUAs) could be used as an alternative source for the production of small diameter vascular grafts. The human umbilical cord (hUC) contains one vein and two arteries [12]. The hUAs are responsible for the transportation of approximately 40 liters of non-oxygenated blood from the fetus to the mother [13]. In addition, their inner diameter is less than 3 mm and their average length (l) could be ranged between 50–60 cm [14]. HUAs are characterized by three distinct layers, tunica intima, media, and adventitia. Endothelial cells (ECs), vascular smooth muscle cells (VSMCs), and perivascular cells (PCs) are located in the tunica intima, media, and adventitia of hUAs, respectively, contributing to various vessels functions [15]. ECs and VSMCs are responsible for vasoconstriction and vasodilation, through eNOs activation, cyclic GMP accumulation, and phosphorylation of protein kinase A (PKA) [16]. Moreover, ECs of tunica intima are forming the endothelium layer, a formation with antithrombogenic properties.

HUAs can be isolated non-invasively from the hUC after the gestation, and then can be submitted to decellularization, in order to eliminate the donor’s cellular populations [17]. The decellularization process can remove the cellular populations of a tissue or organ, while preserving the extracellular matrix (ECM). Moreover, decellularization is a tissue engineering method, which involves physical, chemical, enzymatical methods or a combination of those, to achieve an optimum result [18,19,20]. The preservation of the tissue’s or organ’s ECM is of major importance for ensuring the proper function of the decellularized end product.

Until today, several research groups have used the hUC and its byproducts in several tissue engineering approaches, such as wound healing applications, immunomodulation, bone and cartilage repair [17,21,22]. In the past, the use of a human umbilical vein in peripheral bypass surgeries, was also evaluated, but due to significant drawbacks such as difficulties in handling, lack of elasticity, increased fragility, and poor patency rates, soon its application was stopped [23]. Additionally, the development of tissue-engineered vascular grafts from decellularized hUAs, have also been proposed [4,15,17,22,24]. However, in most of these studies, time consuming decellularization protocols, which can last over a week, have been applied to hUAs [17,23]. These laborious protocols may induce significant alterations to the mechanical properties and protein profile of vessels. This in turn, could result in reduced cell adhesion, during the repopulation process, leading to an impaired function. It is known that the loss of the proper folding of arginine-glycine-aspartic acid (RGD)-binding motifs due to the destruction of a tertiary structure of collagen and fibronectin, can lead to impaired EC adhesion. An ideal scaffold, should retain its initial properties (three-dimensional structure, biochemical and biomechanical properties) and efficiently can be repopulated with the desired cellular populations, in order to repair or replace damaged tissues and organs [8]. In the literature, several methods have been described for obtaining specific cellular populations such as ECs and VSMCs, derived either from tissue biopsy, stem cell differentiation, and utilization of induced pluripotent stem cells (iPSCs)-technology [25,26,27]. However, prolonged decellularization periods or low recellularization efficacy could lead to innate immunity activation, initiation of calcification, and rejection, which could be proved as life-threating for the patients. 

In order to assess the potential of hUAs as an alternative source of small diameter vascular grafts, an extended biomechanical and proteomic evaluation, should be performed. In this study, we introduced a rapid decellularization protocol based on modifications of a previously described protocol from our lab, for the production of tissue engineered vascular grafts derived from hUAs [4,24]. Furthermore, histological and biochemical analysis were performed in decellularized vessels. The evaluation of the mechanical properties of the produced vascular grafts involved the uniaxial testing in circumferential and longitudinal directions. To identify the preserved proteins after the decellularization approach, liquid chromatography (LC) combined with tandem mass spectrometry (MS) was used. The results of this study provide insights on the biomechanical behavior and proteomic profile of the decellularized hUAs, which could serve as small diameter vascular grafts.

## 2. Experimental Section

### 2.1. Isolation of hUAs from Delivered hUCs

HUAs were isolated from hUCs (*n* = 10), that were delivered to the Hellenic Cord Blood Bank (HCBB). HUCs were obtained from end term gestations (38–40 weeks), either with normal or caesarian delivery. Each hUC was accompanied by a signed informed consent by the mother before the gestation. The informed consent was in accordance with the Helsinki declaration and fulfilled the ethical standards of the Greek National Ethical Committee. The overall study has been approved by the Bioethics Committee of BRFAA (No. 7580, 11 October 2018). In this study, only hUCs with gestational days above 38 weeks, were used. The hUCs were delivered to HCBB sooner (less than 36 h) after the gestation and the overall storage time until processing did not exceed 24 h. 

Before the isolation of hUAs, the hUCs were washed with a phosphate buffer saline 1× (PBS 1×, Gibco, Life Technologies, Grand Island, NY, USA), to remove excess blood and blood clots. Then, sterilized forceps and scissors were used for the removal of human umbilical vein, and Wharton’s jelly tissue and finally for the isolation of hUAs. The intact hUAs (*n* = 10, *l* = 5 cm) were used for the next series of experiments. HUAs with entrapped clots in the vessel lumen were excluded, due to possible damage of the vascular wall. Then, each hUA was cut into two parts of equal length. One part served as a native hUA, whereas the other (of the same hUA) was submitted to decellularization. Moreover, the infant’s gender, weight, and gestation days were also recorded in this study. 

### 2.2. Decellularization of hUAs 

HUAs (*n* = 10, length = 2 cm) upon isolation from hUCs, were submitted to decellularization according to a previous described protocol from our lab with some modifications [4,24]. Initially, the hUAs were incubated in a decellularization buffer 1 (DB1), which consisted of 8 mM CHAPS, 1 M NaCl, and 25 mM EDTA in PBS 1× (Gibco, Life Technologies, Grand Island, NY, USA), for 12 h (h) at room temperature (RT). Briefly washing of hUAs for the removal of the excess of DB1 with PBS 1×, was performed. Then, the hUAs were placed in DB2, consisting of 1.8 mM SDS, 1 M NaCl, and 25 mM EDTA in PBS 1× (Gibco, Life Technologies, Grand Island, NY, USA) for another 12 h at RT, followed by briefly washing with PBS 1× (Gibco). Finally, the hUAs were incubated at 37 °C for 12 h in a α-Minimum Essentials Medium (α-ΜΕΜ, Gibco, Life Technologies, Grand Island, NY, USA) with 40% *v*/*v* Fetal Bovine Serum (FBS, Gibco, Life Technologies, Grand Island, NY, USA), ensuring the complete removal of genetic material remnants. All incubation steps were performed under continuous agitation.

### 2.3. Histological Analysis

Histological analysis of hUAs was performed for the evaluation of the decellularization process. Native (*n* = 10) and decellularized (*n* = 10) hUAs, were fixed overnight with 10% *v*/*v* neutral buffered formalin (Sigma-Aldrich, Merck, Darmstadt, Germany), dehydrated, paraffin embedded, and sectioned at 5 μm. For the histological analysis, Hematoxylin and Eosin (H&E, Sigma-Aldrich), Sirius Red (SR, pH = 2, Sigma-Aldrich, Merck, Darmstadt, Germany), and Orcein (OS, Sigma-Aldrich, Merck, Darmstadt, Germany) stains were performed for the evaluation of cellular and nuclear materials, collagen and elastin, respectively. Images were acquired with a Leica DM L2 light microscope (Leica microsystems, Weltzar, Germany) and processed with Image J v. 1.46r (Wane Rasband, National Institutes of Health, Bethesda, MD, USA).

### 2.4. Immunofluorescence of hUAs

Further histological analysis, the indirect immunofluorescence of hUAs against collagen I and fibronectin were involved. Slides of native and decellulized hUAs were deparaffinized, rehydrated, and blocked. The sections were incubated with monoclonal antibodies against collagen I (1:2000, Sigma-Aldrich) and fibronectin (1:200, Sigma-Aldrich, Merck, Darmstadt, Germany), followed by incubation with FITC-conjugated mouse IgG (1:80). In addition, the DAPI stain (Sigma-Aldrich, Merck, Darmstadt, Germany), was added to all samples. Finally, the sections were dehydrated and glycerol mounted. Images were acquired with a Leica SP5 II coupled with LAS Suite v. 2 software (Leica microsystems, Weltzar, Germany).

### 2.5. Scanning Electron Microscopy

Scanning electron microscopy (SEM) analysis was also performed in native (*n* = 3, l = 0.5 cm) and decellularized (*n* = 3, l = 0.5 cm) hUAs. Segments of native and decellularized hUAs were fixed with a 1% *v*/*v* glutaraldehyde solution (Sigma-Aldrich) for 30 min, followed by briefly washing with distilled water. Then, the samples were dehydrated in 70, 80, and 95% *v*/*v* aqueous ethanol and absolute ethanol with 20 min exchanges. The segments of hUAs were incubated for 10 min with hexamethyldisilazane (HMDS, Sigma-Aldrich, Merck, Darmstadt, Germany) and sputter coated with gold (Cressington Sputter, Coater 108 auto, Watford, United Kingdom). Finally, the samples were examined with a Phillips XL-30 SEM (Phillips, FEI, Hillsboro, OR, USA).

### 2.6. Biochemical Analysis of hUAs

Collagen, sGAGs, and DNA quantification was performed in native (*n* = 10, l = 1 cm) and decellularized (*n* = 10, l = 1 cm) hUAs. 

Specifically, the collagen content was estimated based on the quantification of hydroxyproline. For this purpose, the Hydroxyproline Assay kit (MAK008, Sigma-Aldrich, Merck, Darmstadt, Germany) was applied, according to the manufacturer’s instructions. For sGAG quantification, all samples were initially digested with a papain buffer (125 μg/mL, Sigma-Aldrich, Merck, Darmstadt, Germany) at 60 °C for no more than 12 h. Then, dimethylene blue (DMB, Sigma-Aldrich, Merck, Darmstadt, Germany) was added to the digests, followed by a photometrically measurement at 525 nm. The estimation of sGAG concentration in each sample was performed through interpolation to a standard curve. Chondroitin sulfate in a concentration of 12, 25, 50, 100, and 150 μg/mL was used as a standard for the development of the standard curve.

The DNA content was quantified in native (*n* = 10, *l* = 1 cm) and decellularized (*n* = 10, *l* = 1 cm) hUAs samples. Briefly, the samples were digested in a lysis buffer (0.1 mol Tris, 0.2 mol NaCl, 5 mmol NaCl, and 25 mg/mL Proteinase K, Sigma-Aldrich, Merck, Darmstadt, Germany). The incubation time was 12 h at 55 °C with mild agitation, followed by inactivation of Proteinase K at 65 °C for 5 min. The DNA was ethanol cleaned and photometrically measured with Nanodrop (Thermo Scientific, Waltham, MA, USA) at 260 to 280 nm.

### 2.7. Specimen Preparation for Biomechanical Analysis and Geometrical Evaluation

A ring-like (~1-mm width) and a strip-like sample (~5-mm long) were kept for qualitative histological observations. The left-over arterial segment was kept for biomechanical studies, during which it was stored in a Krebs-Ringer solution at 4 °C and slowly warmed to 37 °C before testing. At that time, the vessel was cut into a ring and a strip sample along the circumferential and longitudinal direction, respectively. These were placed inside a Petri dish containing a Krebs-Ringer solution at 37 °C, and their frontal and lateral aspects were photographed with a color digital camera (Leica DFC500; Leica Microsystems GmbH, Wetzlar, Germany) coupled to a stereomicroscope (Nikon SMZ800; Nikon Instruments Europe BV, Amsterdam, The Netherlands). The inner and outer circumference, thickness, cross-sectional area, and width of the ring sample, as well as the length, thickness, and width of the strip sample were measured from the pictures in their unloaded state with the Image-Pro Plus software (v. 4.5, Media Cybernetics Inc, Bethesda, MD, USA). Thickness and width measurements were automatically conducted at a hundred equidistant locations along the circumference of rings and the length of strips, and then by taking the average. 

### 2.8. Uniaxial Tension: Setup and Methods

The experimental device (Vitrodyne V1000 Universal Tester; Liveco Inc, Burlington, VT, USA) consisted of (a) a stationary lower grip and an upper grip attached to the actuator, gradually extending the samples that were vertically mounted in the grips; (b) a load cell (GSO-250; Transducer Techniques, Temecula, CA, USA) with 0.01-g accuracy for the evaluation of load; (c) a rotary encoder providing feedback on the vertical displacement of the upper sample grip with 10-μm accuracy; (d) a saline bath wherein the samples were submerged during testing to sustain normal tissue hydration; (e) a heater coil (1130A, PolyScience, Niles, IL, USA) regulating the temperature of the saline bath at 37 °C; and (f) an accompanying personal computer, interfaced with the controller of the device via the Material Witness software package (v. 2.02, Liveco Inc, Burlington, VT, USA), to store the data. Hook-shaped grips were used for mounting the ring samples and parallel plate grips for the strip samples. Small pieces of non-slip paper were attached to the inner surfaces of the parallel plate grips, thereby providing sufficient friction to avoid sample slippage. The unloaded length of the samples was obtained by adjusting vertically the upper grip to record only their weight and certified by the absence of sample folding. The samples were submitted to a progressively increasing tensile load at a 10-μm/s rate until full rupture of the wall, as in previous studies from our group [28,29]. Unlike the rings, the majority of strips exhibited multiple ruptures. Only data up to the first rupture were evaluated and presented. Synchronized output from the load cell and the rotary encoder was transmitted to the accompanying computer with a 50-Hz sampling frequency. Tissue properties in both directions were examined to determine direction-dependent differences.

### 2.9. Biomechanical Data Processing

Stretch was calculated as the ratio of sample length at each load to the unloaded length and strain as stretch minus one. Stress was calculated by dividing the product of the load and stretch by their unloaded cross-sectional area, assuming tissue incompressibility. The stress-strain data were regressed with 9th-order polynomials, affording correlation coefficients *r* > 0.95, and the elastic modulus (tangent) at each strain level was calculated as the first derivative of stress over strain. Failure stress, representing tissue strength, and failure strain, representing extensibility, were calculated as the maximum stress and strain values at the first rupture. Peak elastic modulus, representing maximum tissue stiffness, was calculated as the highest elastic modulus value before the first rupture. All calculations were semi-automatically performed using a routine programmed in Mathematica (v. 9.0, Wolfram Research Inc, Boston, MA, USA). 

### 2.10. HUAs Preparation for Proteomic Analysis

Native (*n* = 5) and decellularized (*n* = 5) hUAs were digested with a sample buffer (7 M urea, 2 M thiourea, 4% CHAPS, 1% DTE, and 3.6% Protease inhibitors. Sigma-Aldrich, Merck, Darmstadt, Germany). The Bradford assay was performed for the determination of samples concentration. A sample reduction with 10 mM DTE for 20 min at room temperature (RT), was performed. The next step involved the addition of the buffer exchange to the samples, followed by centrifugation at 16,000 g for 15 min. This step was performed in Amicon Ultra Centrifugal filter devices (0.5 mL, 30 kDa MWCO; Merck Millipore). The addition of an urea buffer (8 M urea in 0.1 M Tris-HCl pH 8.5, Merck, Darmstadt, Germany) in all samples was followed. Protein alkylation was performed with 0.05 M iodoacetamide in an urea buffer for 20 min at RT. Briefly washing with an urea buffer (two times) and an ammonium bicarbonate buffer (50 mM NH4HCO3 pH 8.5, Sigma-Aldrich, Darmstadt, Germany) in all samples was performed. Finally, trypsinization of proteins (trypsin ratio 1:100, Sigma-Aldrich, Darmstadt, Germany), centrifugation at 16,000 g for 10 min, and lyophilization were performed.

### 2.11. LC-MS/MS of hUAS

The proteomic analysis of all samples was performed using LC/MS on the Dionex Ultimate 3000 UHPLC system coupled with the high resolution nano-ESI Orbitrap-Elite mass spectrometer (Thermo Scientific). A loading solution of 0.1% *v*/*v* formic acid (Sigma-Aldrich, Darmstadt, Germany) was used for the reconstitution of the samples. The samples were loaded on the Acclaim PepMap 100, 100 μm × 2 cm C18, 5 μm, 100 Ȧ trapping column with the ulPickUp Injection mode with the loading pump operating at a flow rate of 5 μL/min. In order for the peptides of the samples to be separated the Acclaim PepMap RSLC, 75 μm × 50 cm, nanoViper, C18, 2 μm, 100 Ȧ column retrofitted to a PicoTip emitter was used. The mobile phases A and B were composed of 0.1% formic acid (Sigma-Aldrich, Darmstadt, Germany) and 100% acetonitrile (Sigma-Aldrich, Darmstadt, Germany), 0.1% formic acid (Sigma-Aldrich, Darmstadt, Germany), respectively. Then, the elution of peptides for 240 min from 2% to 33% was performed. The flow rate and the temperature of the column were 300 nl/min and 35 °C, respectively. Positive ion electrospray ionization was applied in order to achieve the gaseous exchange transition of the peptides. Top 10 precursor ions between m/z ratio 300 and 2200, intensity threshold 500 counts, and FT mass resolution of 60,000, were subjected in a higher-energy collisional dissociation (HCD) fragmentation. Tandem mass spectra were finally acquired. Normalized collision energy was set to 33 and the precursor ions were excluded for final activation (45 s, 5 ppm mass tolerance).

### 2.12. Protein Identification

Proteome Discoverer 1.4 (Thermo Finnigan) coupled with the Sequest search engine and Uniprot database (Homo sapiens) was used for the analysis of the raw files. Peptide identification was performed taking into consideration the following parameters. (1) Carbamidomethylation of cysteine and oxidation of methionine as static and dynamic modifications. (2) Two missed cleavage sites, a precursor mass tolerance of 10 ppm, and fragment mass tolerance of 0.05 Da were allowed. In addition, the q-value (target False Discovery rate FDR-strict: 0.01, target FDR-relaxed: 0.05) was applied for the FDR validation.

The mass spectrometry proteomics data have been deposited to the ProteomeXchange Consortium via the PRIDE [30] partner repository with the dataset identifier PXD020187 (Appendix A). The classification of the identified proteins was performed using the Panther classification system (www.pantherdb.org). Furthermore, key specific protein associations were obtained, using the STRING v11 protein database. The proteins COL3A1 (P12111), COL1A2 (P12110), and FN1 (PO2751) were subjected to the STRING v11 database to map the canonical pathways and protein networks.

### 2.13. Statistical Analysis

Microcal Origin (v. 9.0; OriginLab^®^ Corp., Northampton, MA, USA) was used for the statistical analysis of the current study. Collagen, sGAG, and DNA quantification data were compared using the Mann-Whitney test. Statistical significance was considered when the *p*-value was less than 0.05. Indicated values were the mean ± standard deviation. The unpaired Student’s *t*-test was used for comparisons of the biomechanical parameters between decellularized and native arteries, while comparisons between directions were assessed with the paired *t*-test. The Pearson correlation coefficient r was used for identifying associations of the biomechanical parameters with gestational age, infant’s weight, and arterial caliber, i.e., wall thickness and diameter. The effect of infant’s gender on the biomechanical properties was assessed with the unpaired *t*-test. 

## 3. Results

### 3.1. Histological Evaluation of hUAs

**Figure 1:**
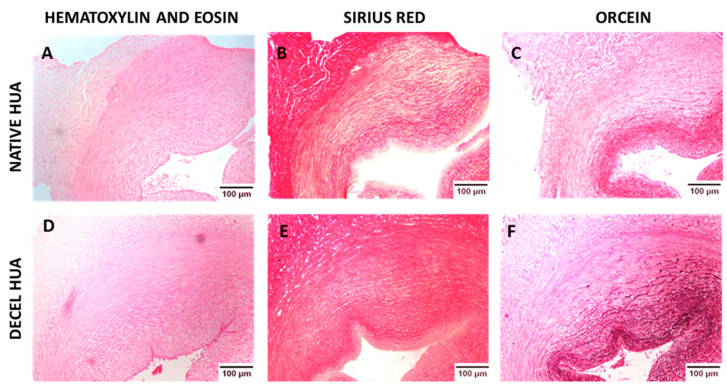
Histological analysis of human umbilical arteries (hUAs). Native hUA stained with Hematoxylin and Eosin (H&E) (**A**), Sirius Red (SR) (**B**), and orcein stain (OS) (**C**). Decellularized (Decel) hUA stained with H&E (**D**), SR (**E**), and OS (**F**). Images were obtained with original magnification 10× and scale bars 100 μm.

**Figure 2:**
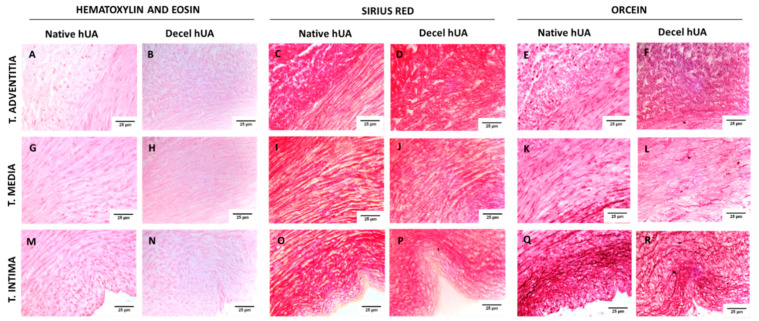
Multilayer histological analysis of human umbilical arteries (hUAs). Tunica adventitia of native and decellularized (decel) hUA stained with Hematoxylin and Eosin (H&E) (**A**,**B**), Sirius Red (SR) (**C**,**D**), and orcein stain (OS) (**E**,**F**), respectively. Tunica media of native and decellularized hUA stained with H&E (**G**,**H**), SR (**I**,**J**), and OS (**K**,**L**), respectively. Tunica intima of native and decellularized hUA stained with H&E (**M**,**N**), SR (**O**,**P**), and OS (**Q**,**R**), respectively. Images were acquired with 40× original magnification and scale bars 25 μm.

HUAs were effectively decellularized with the use of the proposed protocol, as has been indicated by histological analysis. No cellular or nuclear materials were evident in decellularized hUAs stained with H&E stain (Figure 1). Moreover, key ECM components such as collagen and elastin were retained after the decellularization according to SR and OS (Figure 1). Specifically, collagen fibers are distributed in the vascular wall of hUAs in a specific orientation. In tunica intima and media the collagen fibers are mostly circumferentially aligned, whereas in tunica adventitia, the collagen fibers are characterized by a combination of longitudinal and circumferential alignment (Figure 2). In addition, this specific orientation of collagen fibers was preserved in decellularized hUAs (Figure 2). On the other hand, hUAs were characterized by a few circumferentially oriented elastin fibers, localized mostly to tunica intima. Tunica media contains a few elastin fibers, whereas no elastin fibers were observed in tunica adventitia. Elastin fibers with the same orientation were preserved in decellularized hUAs, according to the OS results (Figure 2).

**Figure 3:**
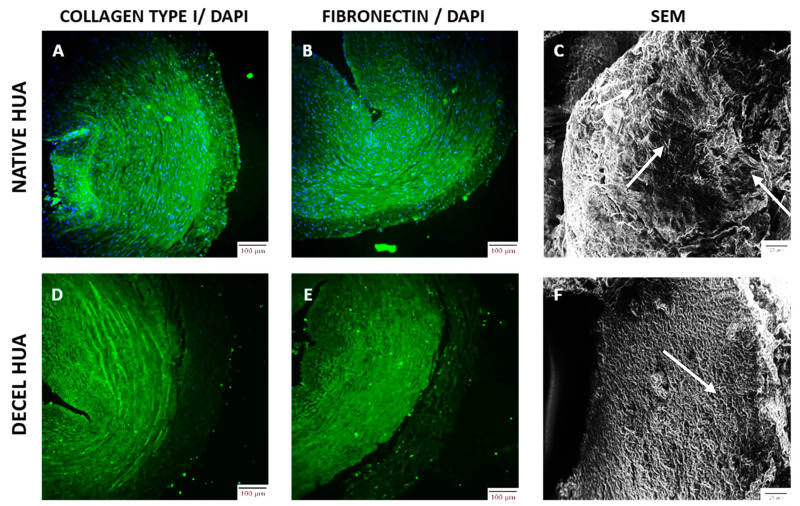
Indirect immunofluorescence and scanning electron microscopy (SEM) analysis in human umbilical arteries (hUAs). Indirect immunofluorescence against collagen type I (**A**,**D**) and fibronectin (**B**,**E**) in combination with DAPI staining in native and decellularized (decel) hUAs, respectively. Blue spots in images A and B represent the presence of cell nuclei. Images were obtained with original magnification 10× and scale bars 100 μm. SEM analysis of native (**C**) and decellularized (**F**) hUAs. White arrows in image C, indicate the presence of cellular populations. White arrows in image F, indicate the preservation of ECM proteins, while no cellular populations are evident. Images were obtained with original magnification 40× and scale bars 25 μm.

Indirect immunofluorescence analysis indicated the presence of collagen type I and fibronectin in the vascular wall of the vessels, which were successfully preserved after the decellularization process (Figure 3, Figures S1 and S2). No DAPI stain was evident in decellularized hUAs, suggesting the successful removal of cellular and nuclear materials (Figures S1 and S2). Moreover, SEM images revealed the distribution of cellular populations to the entire native hUAs (Figure 3). Decellularized hUAs were characterized by the elimination of these cellular populations, while the ECM was preserved intact (Figure 3). Indirect immunofluorescense and SEM images, further confirm the initial histological analysis, indicating the successful decellularization of hUAs. 

### 3.2. Biochemical Analysis and DNA Quantification

**Figure 4:**
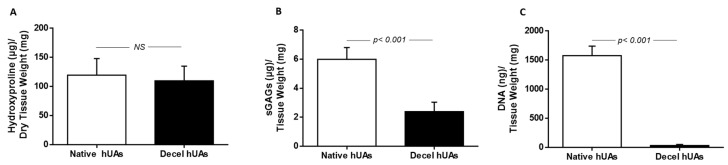
Biochemical and DNA content evaluation of hUAs before and after the decellularization process. Total hydroxyproline content (representing collagen content), sGAGs content, and DNA quantification of native and decellularized hUA (**A**–**C**), respectively. Statistically significant differences were observed in the sGAGs (*p* < 0.001) and DNA (*p* < 0.001) content between native and decellularized hUAs.

Biochemical analysis of the hUAs involved the quantification of total hydroxyproline content (which corresponds to the collagen content) and sGAGs content. The hydroxyproline content was decreased in decellularized hUAs (110 ± 24 μg hydroxyproline/mg dry tissue weight) compared to native hUAs (119 ± 27 μg hydroxyproline/mg dry tissue weight), but this decrease was not statistically significant (*p* < 0.05, Figure 4). On the other hand, a statistically significant difference was observed in the sGAGs content between native and decellularized vessels (*p* < 0.01). Specifically, the sGAGs content before the decellularization was 6 ± 1 μg sGAG/mg dry tissue weight, and after the decellularization was 2 ± 1 μg sGAG/dry tissue weight (Figure 4). The DNA content appeared to significantly decrease in decellularized hUAs. The DNA amount of native and decellularized hUAs was 1577 ± 158 and 33 ± 5 ng DNA/mg dry tissue weight, respectively (Figure 4).

### 3.3. Biomechanical Analysis of hUAs

**Figure 5:**
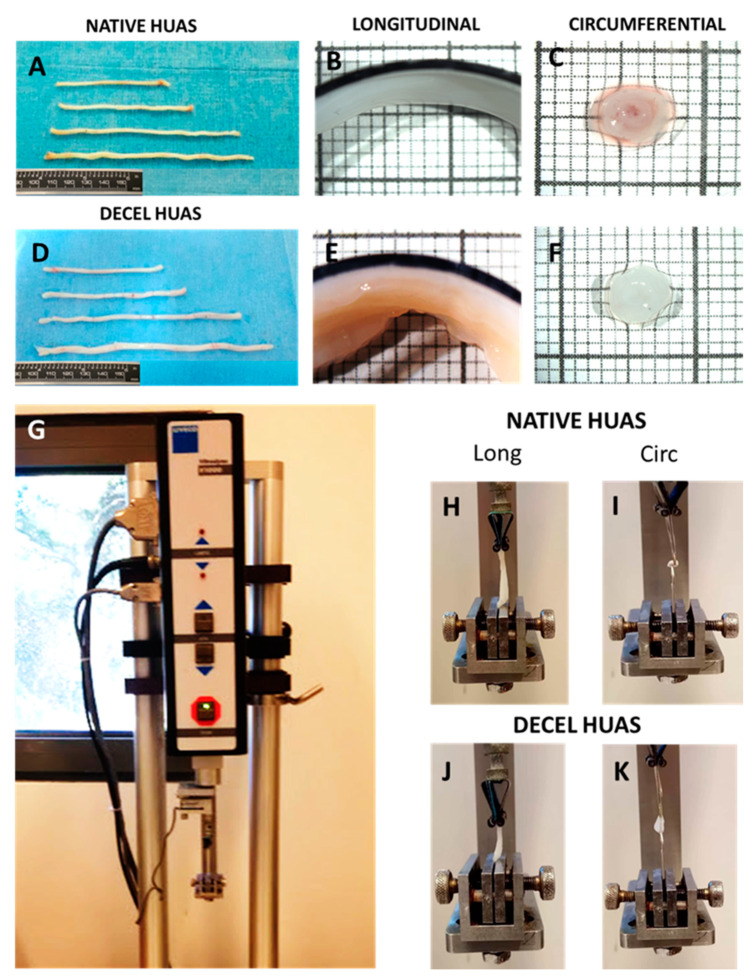
Biomechanical analysis of human umbilical arteries (hUAs). Overview of native and decellularized (decel) hUAs (**A**,**D**). HUAs segments in longitudinal (log) (**B**,**E**) and circumferential (circ) direction (**C**,**F**). Vitrodyne uniaxial testing machine, where the biomechanical analysis of hUAs, was conducted (**G**). Uniaxial testing of native and decellularized hUAs segments in longitudinal (**H**,**J**) and circumferential (**I**,**K**) directions.

The stress-strain behavior of hUAs was determined with the performance of uniaxial biomechanical testing. For this purpose, native and decellularized hUAs were tested both in a longitudinal and circumferential direction (Figure 5). 

**Figure 6:**
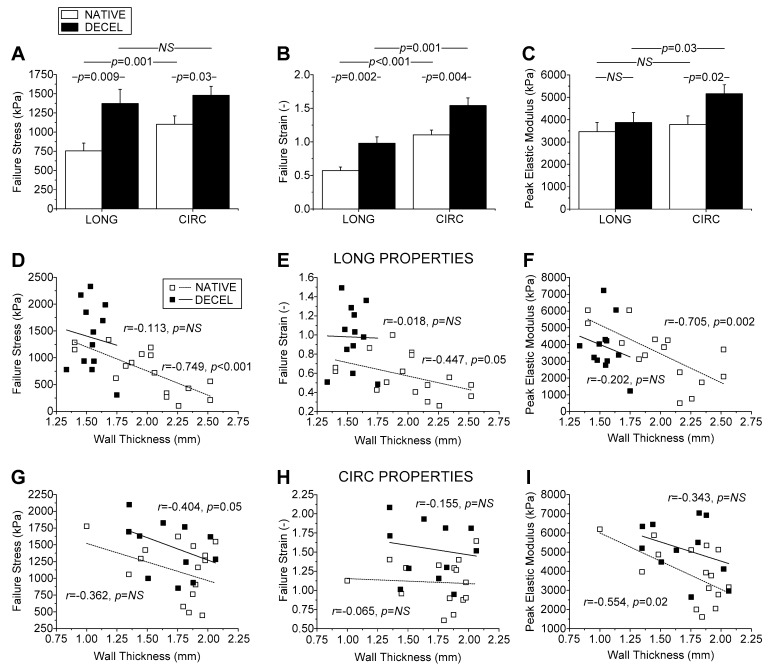
Uniaxial biomechanical testing of hUAs. Failure stress (**A**), failure strain (**B**), and peak elastic modulus (**C**) of native and decellularized hUAs. Correlation of failure stress (**D**), failure strain (**E**), and peak elastic modulus (**F**) with wall thickness in a longitudinal direction. Correlation of failure stress (**G**), failure strain (**H**), and peak elastic modulus (**I**) with wall thickness in a circumferential direction.

Specifically, in a longitudinal direction the maximum stress (σ), peak elastic modulus, and maximum strain for native and decellularized vessels were 755 ± 393 and 1373 ± 566 kPa, 3458 ± 1601 and 3867 ± 1141 kPa, 1.4 ± 0.1 and 1.7 ± 0.2 kPa, respectively (Figure 6). Accordingly, in circumferential direction the maximum stress (σ), peak elastic modulus, and maximum strain for native and decellularized vessels were 1102 ± 426 and 1480 ± 388 kPa, 3781 ± 1476 and 5153 ± 1368 kPa, 2.1 ± 0.3 and 2.7 ± 0.6 kPa, respectively (Figure 6). Statistically significant differences in longitudinal direction between native and decellularized hUAs were observed only in maximum stress (p < 0.013) and strain (*p* < 0.001). On the other hand, all parameters tested for circumferential direction exhibited statistically significant differences (*p* < 0.05) between the two study groups. In both artery types, greater parameter values were found circumferentially than longitudinally. Figure 6 shows the relationships between the failure parameters and wall thickness, noting that similar relationships were acquired when considering associations with the diameter. All parameters were inversely related to thickness, with the correlation coefficients being moderate to strong and significant in several occasions. At each thickness level, the failure stress and failure strain values of decellularized arteries were higher than those of native arteries in either direction, and the same was evidenced in regards to the peak elastic modulus yet only circumferentially, providing further validation to the bar charts shown in Figure 6. Non-significant and weak were the associations found between the failure parameters and the gestational age and infant’s weight, and the infant’s gender had a non-significant effect (Figure 6 and Figure S3).

### 3.4. Proteomic Evaluation of hUAs

Proteomic analysis of hUAs was conducted in order to further evaluate the efficacy of the current decellularization protocol. For this purpose, LC/MS was performed in hUAs before and after the application of the decellularization method. In total, 1109 ± 20 and 801± 68 proteins were identified in native and decellularized hUAs, respectively. Representative data with the 50 most abundant identified proteins in both samples are presented in Table 1 and Table 2. Detailed description and the total number of identified proteins are provided as supplementary material (Tables S1–S10). Among them, ECM, cytoskeletal, structural, and enzymes were identified both in native and decellularized hUAs. Based on the Panther online tool, molecular function classification of the identified proteins of native and decel hUAs, was performed. Specifically, the classification involved proteins with (1) translation regulator activity, (2) transcription regulator activity, (3) molecular transducer activity, (4) binding, (5) structural molecule activity, (6) molecular function regulator, (7) catalytic activity, and (8) transporter activity, as shown in Table 3.

Based on the STRING database analysis, interactions between ECM proteins (preserved in decellularized hUAs) including collagen alpha-3 (VI) chain, fibronectin, collagen alpha-2 (VI) chain, and laminin subunit alpha-1 with cell adhesion molecules such as α1β1, α2β1, α5β1, and α5β8, were shown (Figure S4). These molecules are expressed in a wide variety of cellular populations, such as ECs and VSMCs, indicating further their possible adhesion to the produced acellular matrix, upon repopulation.

## 4. Discussion

Reconstructive cardiovascular surgery requires the use of suitable biomaterials as substitutes for the damaged or obstructed vessels [3]. On the contrary to large diameter vascular grafts (inner diameter >6 mm), where synthetic biodegradable conduits are applied successfully, the reconstruction of small diameter vascular grafts requires advanced tissue engineering approaches [3,4,5,6]. The majority of patients with CAD (>60%) lack suitable autologous vessels. In this case, synthetic conduits made from ePTFE or Dacron are mostly used. Despite the significant adverse reactions, which have been observed, currently their use has received FDA approval [3]. As a result, the success rate of the synthetic grafts within the first year of implantation is limited to 60%, due to mismatch complications, intima hyperplasia, and initiation of the calcification process [7]. Additionally, the use of animal derived vessels (mostly bovine and porcine vessels) still face significant difficulties to their application, due to the presence of a-gal epitopes. The presence of these epitopes can lead to immune system activation by the host, thus resulting in their rejection [18]. Given that, reconstructive surgery has gained great attention by the scientific society, alternative strategies may be used.

Taking into consideration the above data, the hUAs may represent significant candidates for reconstructive vascular surgery. HUAs are small diameter muscular vessels with an inner diameter less than 3 mm, and are responsible for the transportation of non-oxygenated blood from the fetus [13,14]. In the past, hUAs were successfully decellularized, with the use of different protocols. However, the time needed for the proper decellularization, may be proven significant for its further application as a tissue engineered vascular graft. Therefore, the aim of this study was the evaluation of a rapid decellularization approach in hUAs, in order to be used for the development of acellular vascular conduits. For this purpose, histological and biochemical analysis was conducted before and after the proposed decellularization method. To evaluate better the effect of the decellularization process in hUAs, biomechanical and a wide proteomic analysis, using LC/MS was also performed.

To begin with, histological analysis was performed in native and decellularized hUAs, using H&E, SR, and OS. Specifically, we observed no cellular or nuclear materials in the decellularized hUAs, while at the same time the vessel’s ECM was properly preserved. SEM analysis revealed also the preservation of vessel’s ECM and the absence of cellular populations in decellularized hUAs, confirming further the above results. Further histological analysis with SR and OS confirmed the presence of collagen and elastin fibers in the decellularized vessels. Moreover, the alignment of collagen and elastin fibers was retained in all vessel’s layers (tunica intima, media, and adventitia) in the decellularized hUAs. The proper orientation of key ECM proteins is of great importance for the vessel function (contraction and relaxation) [31]. Importantly, the preservation of key ECM components such as collagen and elastin in the decellularized hUAs is of major importance. In the future, advanced approaches such as the near-infrared (NIR) laser light, second harmonic generation (SHG), and two photon excited fluorescence (TPF), can add significant information regarding the collagen and elastin microstructure [32]. The SHG method is based on the nonabsorptive, nonlinear light interaction with collagen fibrils, revealing in this way important parameters of collagen structure. On the other hand, TPF relies on the excitation of fluorophores, generated by pyridinium cross-links. When these methods are combined, additional information regarding the collagen and elastin microstructure such as triple helix, molecular packing and in general their structural organization [32].

Additionally, decellularized vascular grafts should also retain their cell adhesion properties. Indeed, the preservation of collagen and elastin fibers alignment, may also indicate the proper preservation of RGD binding motifs in those proteins. Collagen and elastin fibers are characterized by a great number of RGD binding motifs, promoting further the adhesion of specialized cellular populations such as ECs and vascular VSMCs [31]. The adhesion properties of these cellular populations are exerted through the interaction of integrins (α1β1, α2β1, and aνβ1) with the RGD binding motifs of collagen and elastin fibers [33,34,35]. Indirect immunofluorescence confirmed the presence of collagen I and fibronectin, which are important structural proteins of the vessel wall. It has been shown, that fibronectin may promote and retain the CD34+ CD31+ EC differentiation status, through the Wnt canonical signaling pathway [36]. The above results seemed to be consistent with previous work from our group, although a different decellularization approach was used [24]. The next step of this study involved the biochemical evaluation of the hUAs. Specifically, it was reported that a decrease in the hydroxyproline content between native and decellularized hUAs, was not statistically significant. Decellularized hUAs were characterized by lower sGAGs and DNA content compared to native vessels. Moreover, these differences in the DNA and sGAGs content were statistically significant. It is known that sGAGs can interact with a “core” protein, resulting in formation of large macromolecules, known as proteoglycans. Proteoglycans such as chondroitin and dermatan sulfate, are heavily glycosylated proteins and are responsible for the alignment mostly of the collagen fibers [37]. The loss of proteoglycans in decellularized hUAs may cause significant alterations to collagen fibers orientation, resulting in modification of the vessel’s function properties. On the other hand, based on the histological analysis, no modification in collagen and elastin fibers orientation was observed in the vascular wall of the study vessels. In regard to the DNA content, decellularized hUAs were characterized by significantly lower content compared to initial vessels, which is consistent with the previous studies. Moreover, Crapo et al. proposed that a properly decellularized ECM may be characterized by no visible of cellular and nuclear materials (observed either with H&E or DAPI) and by <50 ng/mg ECM dry tissue [38]. Both criteria are fulfilled in the current study, indicating further the successful decellularization of hUAs with the proposed protocol.

Biomechanical testing revealed certain differences in failure parameters between native and decellularized hUAs, suggesting that the latter were significantly stronger and more extensible than the former in either direction, and they were also significantly stiffer albeit only circumferentially. However, differences in mechanical properties of hUA segments used in this study, may also exist due to different anatomical locations in the umbilical cord, which was selected for the initial excision. To address the issue of anatomical dependence on the biomechanical properties of the tissue, we performed correlation analysis. This analysis revealed that all failure parameters, especially those of native arteries, were inversely correlated to wall thickness and arterial diameter. Importantly, the failure parameters of decellularized arteries were greater than those of native arteries at each thickness, justifying us to conclude that the differences found were independent of the anatomical location of the artery tested. 

Overall, the increased strength of decellularized wall, irrespective of gestational age, infant’s weight and gender, and arterial caliber, was consistent with the collagen content in the decellularized than native arteries found biochemically and verified through our qualitative microscopic observations, and similarly holds for maximum tissue stiffness. That the extensibility was also significantly greater in decellularized arteries may be related to the more disorganized fiber arrangement. However, no disorganization either for collagen or elastin fibers was observed in decellularized hUAs (based on histological analysis), unlike other previously performed studies in similar vessels. Another fact that should be noted is that decellularized hUAs are characterized by acellular content. Indeed, ECs and VSMCs are playing major role in vessel function and orientation of collagen and elastin fibers. More importantly, and based on the microscopical images, the collagen and elastin fibers of native hUAs are fully crimped (due to the presence of ECs and VSMCs), whereas the fibers in decellularized vessels were fully uncrimped. In addition, decellularized hUAs exhibited the same amount of collagen but significantly lower amount in sGAG content. The reduction of sGAG content in decellularized vessels could reduce the crimp of the collagen fibers, which further may result in a crosslink of collagen and elastin fibers. Possibly, the above reasons may be responsible for the stiffer behavior of decellularized vessels, which was demonstrated during the biomechanical analysis. Note too that the data discussed above pertains to just the first failure point, although the stress-strain curves of strips exhibited two and sometimes three ruptures in either artery type, unlike the curves of rings that exhibited a single failure point. This is attributable to the composite multilayered structure of the arterial wall, presenting longitudinally-oriented fibers at the inner and outer wall, with circumferentially-oriented fibers in-between, observed here and documented by Sexton et al. [12] and Stehbens et al. [39]. Fibers orthogonally-oriented to the tensile direction did not bear loads, with the rings thus suffering a single rupture of their medial fibers and the strips suffering multiple ruptures of their inner and outer wall fibers that likely failed at different instances.

To evaluate better the effect of the proposed decellularization protocol in the molecular composition of hUAs, a thorough proteomic analysis was performed. In this way, on average 1109 ± 20 proteins for the native and 801 ± 68 proteins for the decellularized hUAs were successfully identified. Among them, the ECM and cytoskeletal proteins were the most abundant in both samples. Indeed, identified ECM proteins included collagen alpha-3 (VI) chain, collagen alpha-2 (VI) chain, collagen alpha-1 (XII) chain, fibronectin, basement membrane-specific heparin sulfate proteoglycan core protein, byglycan, mimecan, etc. The above proteins are essential for the maintenance of tissue ultrastructure. To further support the above observation, the amount of the structural proteins was higher in decellularized hUAs compared to the native samples. On the other hand, the amount of proteins with molecular transducer, transporter, and molecular function regulator activity was lower in the decellularized hUAs compared to the native hUAs, indicating the successful cell removal. These key structural proteins form an acellular matrix, promoting further the cell adhesion, proliferation, and differentiation. To this direction, further analysis of ECM proteins with the STRING database, indicated interactions with cell adhesion molecules such as the integrins, establishing in this way a decellularized matrix capable of cell repopulation. Moreover, the presence of ECM proteins in decellularized vessels, further confirmed the histological results and indicated the success of the current decellularization protocol. 

Moreover, cytoskeletal proteins including actin, aortic smooth muscle, filamin-A, vimentin, tropomyosin alpha-4 chain, myosin-10, transgelin, alpha enolase, etc., were identified in both samples. Despite the great number of identified cytoskeletal proteins in decellularized hUAs, these proteins do not bear antigenicity [17,24]. Taking into consideration the aforementioned results, which indicated the absence of cellular populations, the decellularized hUAs could possibly serve as an acellular matrix without eliciting the host’s immune response. Moreover, this hypothesis is further supported by the fact that human leukocyte antigen (HLA) class I and II molecules were identified only in native hUAs. HLA class I (A, B, and C) and class II (DP, DQ, DR, DM, DOA, and DOB) and genes are located at the short arm of chromosome 6 (6p21) [40,41]. Typically, HLA class I are expressed in all cells and tissues, whereas, HLA class II are expressed in specific cells such as dendritic cells, mononuclear cells, endothelial and epithelial cells. [38,39]. Both HLA molecules play a major role in the immune system, and their expression is related to the antigen presentation and recognition [40,41]. Specifically, in native hUAs the HLA class I histocompatibility antigen, A-68 alpha chain and HLA class II histocompatibility antigen, DRB1-15 beta chain were identified. On the other hand, none of these proteins were identified in decellularized hUAs, indicating further the production of non-immunogenic vascular grafts. In the same way, Quint et al. reported the successful removal of Major Histocompatibility Complex (MHC) class I after the application of decellularization approach in fabricated vessels [42]. Overall, our proteomic results are consistent with previous work from our lab, using 2D gel electrophoresis [24]. However, with the current proteomic approach we were able to identify a greater number of proteins, gathering more data to better understand the matrix composition of the produced vascular grafts. Currently, only a few studies report proteomic analysis results in hUAs [17,24]. In this way, the current study may add valuable information and improve our understanding of the impact of decellularization in vessels.

Suitable tissue engineering vascular grafts (TEVGs) for reconstructive cardiovascular surgery should fulfill a number of design criteria [3,6]. The produced vascular grafts should support the blood flow and withstand intraluminal pressures. Moreover, the grafts should be suitable in length and diameter and characterized by similar mechanical properties with the replaced vessel. Furthermore, the entire graft should be nontoxic and support the adhesion and proliferation either of stem cells or specialized cellular populations such as ECs and VSMCs. Moreover, hemocompatibility tests involving the platelet adhesion assay, hemolysis test, coagulation assay, and antithrombin trial must be performed, before the graft implantation. Tissue engineered vascular grafts must be characterized by low platelet adhesion, to avoid thrombus formation and lumen occlusion [43]. To explore better the initiation of coagulation cascade, the antithrombin trial by measuring the activated partial thromboplastin time (APTT) and thromboplastin time (TT), may add significant information. In addition, the TEVGs may be characterized by hemocompatibility with the recipient’s blood. Indeed, red blood cell lysis below 5% is acceptable for the most of the grafts, serving as small diameter vessel conduits [43]. By fulfilling these criteria, the developed graft should avoid the manifestations accompanied by their implantation, such as intima hyperplasia, activation of immune system, thrombus formation, calcification, and even rejection [3,6]. To this direction, in vivo implantation of decellularized hUAs in small animal models, has been described in the literature by few research teams [17]. Gui et al. implanted for the first time the decellularized hUA as abdominal aorta interposition graft in nude rats [17]. Due to the lack of uniform endothelium layer, the lumen of the graft was occluded within eight weeks. However, after eight weeks of implantation the graft was recellularized with VSMCs as it was indicated by positive staining of α-SMA. In another study performed by Kerdjoudj et al., a different approach in cryopreserved de-endothelialized hUAs was selected, in order to have a better outcome [44]. Specifically, in this approach, multilayers of poly(styrene sulfonate)/poly(allylamine hydrochloride), were developed, to avoid the platelet aggregations and clot formation. Finally, these grafts were served as carotid artery bypass grafts in rabbits. Doppler ultrasound and histological analysis results confirmed that the grafts remained patent for at least 12 weeks after the bypass surgery. Furthermore, successful recellularization of the grafts with VSMCs was also observed [44]. 

The use of decellularized hUAs may represent an alternative solution for bypass grafting of small diameter vessels, however, more research is needed to be performed towards this direction. Specifically, to evaluate better the functionality of decellularized hUAs as small diameter vessels substitutes, in vivo implantation to large animal models must be performed.

Another important parameter is the time needed for the development of the vascular grafts. Currently a great number of decellularization protocols, which are utilizing different reagents and incubation times, are used [18]. Regarding the hUAs, most published protocols required over four working days (96 h) to produce acellular grafts [17]. To this direction, our modified protocol needed only 36 h to achieve the same outcome. This in turn may be related to the better preservation of the ECM key components, thus leading to improved vessel functionality and biocompatibility.

## 5. Conclusions

To conclude, in this study a modified rapid decellularization protocol was applied efficiently to hUAs. The produced grafts were characterized by a similar matrix composition and biomechanical properties, as others from previous published studies. This cost-effective and fast protocol may further be applied to other tissues and organs, with some modifications. The next step of the current study will be the development of a rapid and effective recellularization approach utilizing mostly the ECs. A recent metanalysis conducted by Skovrind et al. [45], reported that there is no immediate benefit to the patency from recellularized vascular grafts with VSMCs (with or without ECs). For this purpose, our future studies will focus on the production of endothelialized TEVGs, and to assess their functionality as grafts by implantation to animal models. The ultimate goal is the production of ready to use vascular grafts utilizing the patient’s own cells. Moreover, the long term storage of personalized vascular grafts may be more feasible utilizing the vitrification approach. In the future, the stored vascular grafts will be used upon demanding, without any time lost. HUAs may represent an alternative valuable source for the production of small diameter vascular grafts, aiming to the personalized medicine. 

## Figures and Tables

**Table 1:** List of representative identified proteins in native human umbilical arteries (hUAs).

No.	Accession Number	Description	Score	% Sequence Coverage
1	P62736	Actin, aortic smooth muscle	2310	57
2	P21333	Filamin-A	1670	50
3	P12111	Collagen alpha-3 (VI)	1106	35
4	P60709	Actin, cytoplasmic 1	1042	53
5	P35749	Myosin-11	877	34
6	A5A3E0	POTE ankyrin domain family member F	816	6
7	P98160	Basement membrane-specific heparan sulfate proteoglycan core protein	632	33
8	P02751	Fibronectin	534	40
9	P17661	Desmin	450	47
10	P12814	Alpha-actinin-1	444	51
11	Q9Y490	Talin-1	436	37
12	O43707	Alpha-actinin-4	396	59
13	P35579	Myosin-9	362	29
14	P09493	Tropomyosin alpha-1 chain	338	24
15	P08670	Vimentin	331	34
16	Q14315	Filamin-C	330	32
17	P67936	Tropomyosin alpha-4 chain	319	17
18	P12109	Collagen alpha-1 (VI) chain	318	33
19	P18206	Vinculin OS = Homo sapiens	316	36
20	P07951	Tropomyosin beta chain	307	17
21	Q09666	Neuroblast differentiation-associated protein AHNAK	293	26
22	P12110	Collagen alpha-2 (VI) chain	260	19
23	P69905	Hemoglobin subunit alpha	257	77
24	Q05707	Collagen alpha-1 (XIV) chain	244	28
25	P46940	Ras GTPase-activating-like protein IQGAP1	233	41
26	O15061	Synemin	232	39
27	P35580	Myosin-10	216	21
28	Q15149	Plectin	214	16
29	Q01995	Transgelin	206	53
30	P02768	Serum albumin	198	37
31	P01023	Alpha-2-macroglobulin	188	41
32	P04406	Glyceraldehyde-3-phosphate dehydrogenase	186	61
33	Q16363	Laminin subunit alpha-4	173	17
34	P21980	Protein-glutamine gamma-glutamyltransferase 2	172	42
35	Q14204	Cytoplasmic dynein 1 heavy chain 1	171	14
36	Q71U36	Tubulin alpha-1A chain	166	50
37	P07437	Tubulin beta chain	160	64
38	P51911	Calponin-1	150	54
39	P20774	Mimecan	149	30
40	P21810	Biglycan	146	32
41	Q15582	Transforming growth factor-beta-induced protein ig-h3	145	34
42	Q99715	Collagen alpha-1 (XII) chain	142	19
43	Q00610	Clathrin heavy chain 1	141	23
44	P54652	Heat shock-related 70 kDa protein 2	140	43
45	P06733	Alpha-enolase	140	46
46	P68371	Tubulin beta-4B chain	138	60
47	P12277	Creatine kinase B-type	136	68
48	P14543	Nidogen-1	135	35
49	P06396	Gelsolin	135	27
50	P08133	Annexin A6 OS	133	39

**Table 2:** List of representative identified proteins in decellularized hUAs.

No.	Accession Number	Description	Score	% Sequence Coverage
1	P62736	Actin, aortic smooth muscle	5114	63
2	P21333	Filamin-A	1644	48
3	P60709	Actin, cytoplasmic 1	1355	57
4	P12111	Collagen alpha-3 (VI) chain	1284	37
5	Q562R1	Beta-actin-like protein 2	970	18
6	P98160	Basement membrane-specific heparan sulfate proteoglycan core protein	891	40
7	A5A3E0	POTE ankyrin domain family member F	873	7
8	P35749	Myosin-11	800	32
9	P02751	Fibronectin	525	38
10	P12109	Collagen alpha-1 (VI) chain	477	36
11	P17661	Desmin	452	51
12	P35579	Myosin-9	397	29
13	Q14315	Filamin-C	366	36
14	P08670	Vimentin	338	33
15	Q9Y490	Talin-1	314	39
16	P35580	Myosin-10	302	26
17	P12110	Collagen alpha-2 (VI) chain	291	22
18	Q14204	Cytoplasmic dynein 1 heavy chain 1	262	22
19	P46940	Ras GTPase-activating-like protein IQGAP1	242	43
20	Q15149	Plectin	238	19
21	Q71U36	Tubulin alpha-1A chain	219	60
22	P21980	Protein-glutamine gamma-glutamyltransferase 2	200	47
23	O15061	Synemin	197	37
24	P07437	Tubulin beta chain	190	67
25	P68371	Tubulin beta-4B chain	179	64
26	O15230	Laminin subunit alpha-5	176	14
27	Q15063	Periostin	174	42
28	P20774	Mimecan	170	30
29	Q13885	Tubulin beta-2A chain	161	64
30	P68366	Tubulin alpha-4A chain	159	38
31	P68104	Elongation factor 1-alpha 1	159	46
32	Q16363	Laminin subunit alpha-4	155	16
33	P12814	Alpha-actinin-1	154	37
34	Q00610	Clathrin heavy chain 1	149	27
35	P21810	Biglycan	146	39
36	Q99715	Collagen alpha-1 (XII) chain	145	18
37	P04406	Glyceraldehyde-3-phosphate dehydrogenase	145	52
38	O43707	Alpha-actinin-4	139	39
39	P55268	Laminin subunit beta-2	138	16
40	P39060	Collagen alpha-1 (XVIII) chain	137	14
41	P14543	Nidogen-1	136	38
42	P06733	Alpha-enolase	135	46
43	P04792	Heat shock protein beta-1	131	62
44	Q05707	Collagen alpha-1 (XIV) chain	131	26
45	P24821	Tenascin OS = Homo sapiens	131	23
46	P51911	Calponin-1 OS = Homo sapiens	128	54
47	O75083	WD repeat-containing protein 1	125	49
48	Q9NZN4	EH domain-containing protein 2	123	50
49	P11532	Dystrophin	115	14
50	P69905	Hemoglobin subunit alpha	115	58

**Table 3:** Classification of the identified proteins in native and decellularized hUAs. Protein classification based on their molecular function was performed using the Panther analysis tool.

No.	Protein Classification	Native hUA (% Presence)	Decel hUA (% Presence)	*p*-Value
1	Translation regulator activity (GO:0045182)	1.4 ± 0.2	1.9 ± 0.2	0.01
2	Transcription regulator activity (GO:0140110)	1.7 ± 0.1	1.8 ± 0.2	0.19
3	Molecular transducer activity (GO:0060089)	3.3 ± 0.4	0.9 ± 0.1	<0.01
4	Binding (GO: 0005488)	39.3 ± 1.2	44.5 ± 2.6	0.04
5	Structural molecule activity (GO:0005198)	4.7 ± 0.5	8.8 ± 0.6	<0.01
6	Molecular function regulator (GO:0098772)	5.7 ± 0.7	2.7 ± 0.5	0.02
7	Catalytic activity (GO:0003824)	36.8 ± 1.6	35.2 ± 1.4	0.26
8	Transporter activity (GO:0005215)	7.1 ± 0.8	4.2 ± 0.7	0.02

## Supplementary Materials

Supplementary materials can be found at https://www.mdpi.com/2227-9059/8/8/280/s1.

## Appendix A

The mass spectrometry proteomics data have been deposited to the ProteomeXchange Consortium via the PRIDE partner repository with the dataset identifier PXD020187. The name of the project that has been deposited in the PRIDE repository is “Proteomic evaluation of decellularized human umbilical artery”.
