# Supplementary material for: Insights into Biomechanical and Proteomic Characteristics of Small Diameter Vascular Grafts Utilizing the Human Umbilical Artery
Source: Biomedicines. 2020 Aug 10;8(8):280. doi: 10.3390/biomedicines8080280 (PMC7460081; doi:10.3390/biomedicines8080280)
Supplement: Supplementary file 1 [file biomedicines-08-00280-s001.zip › Supplementary files/Supplementary files v2.docx]

**
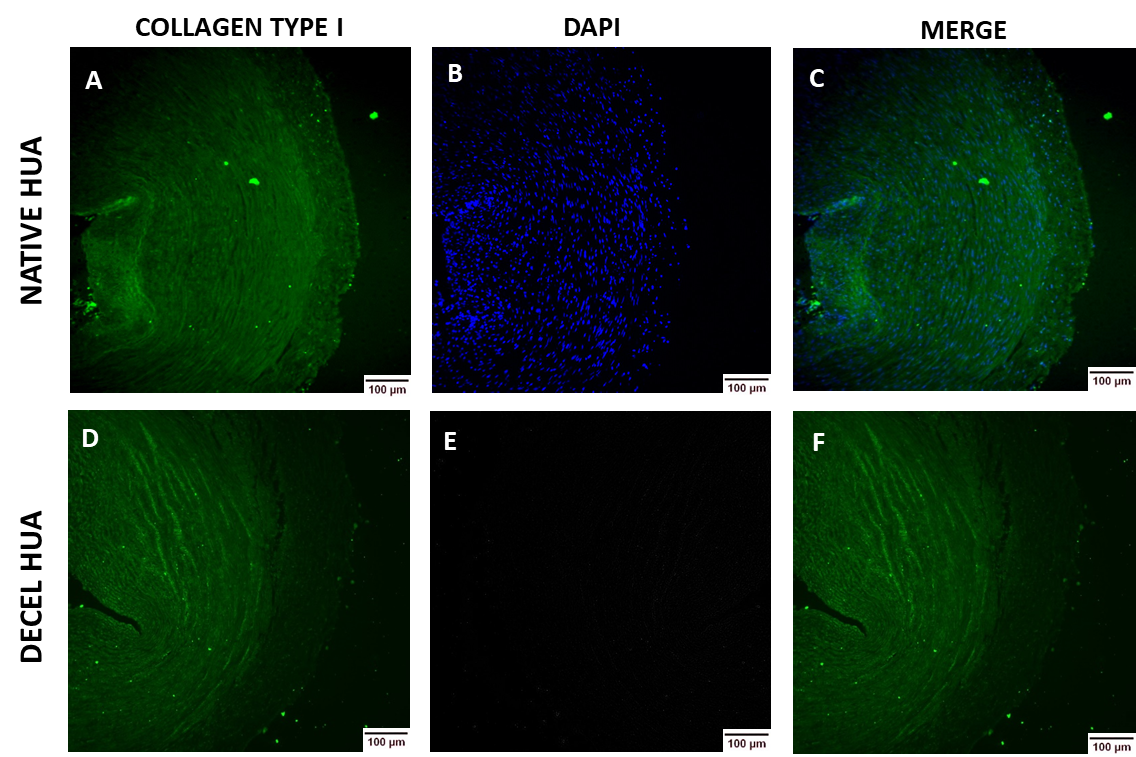
**

**Figure S1.** Indirect immunofluorescence against collagen type I in combination with DAPI in native and decellularized hUAs. Collagen type I (A, D), DAPI staining (B, E) and their combination (C, F) of native and decellularized hUAs. Original magnification 10x, scale bars 100 μm.

**
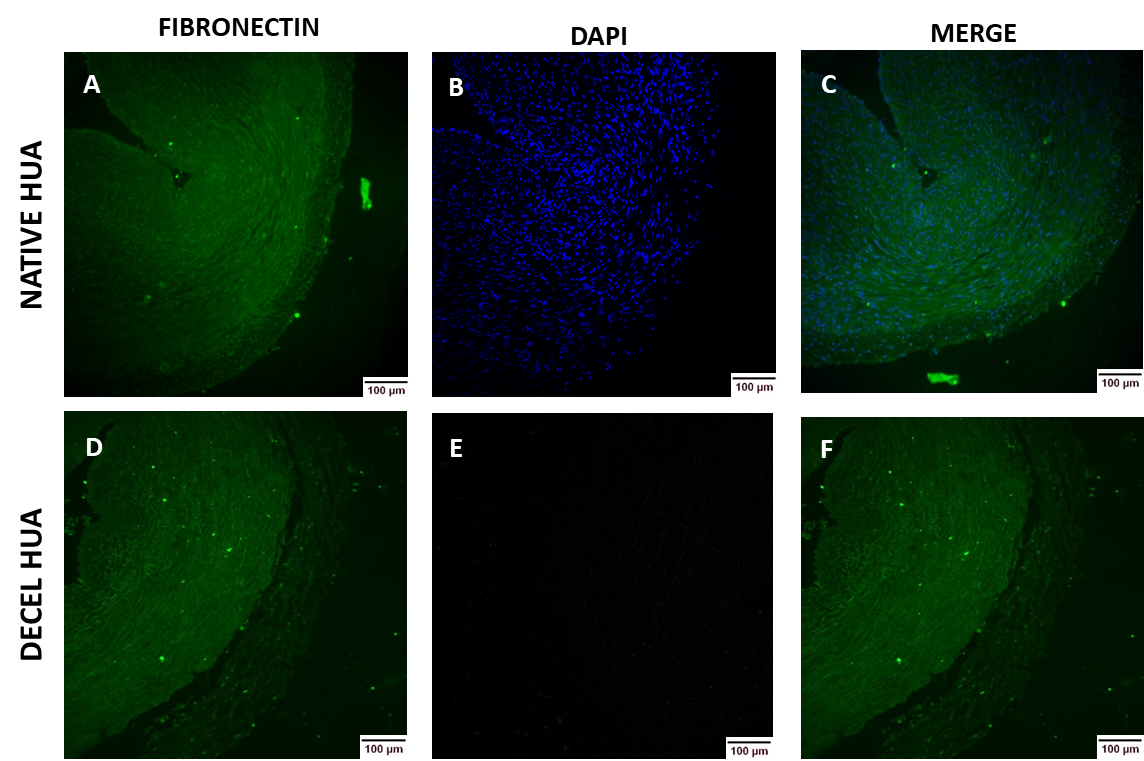
**

**Figure S2.** Indirect immunofluorescence against fibronectin in combination with DAPI in native and decellularized hUAs. Fibronectin (A, D), DAPI staining (B, E) and their combination (C, F) of native and decellularized hUAs. Original magnification 10x, scale bars 100 μm.

| **No** | **Infant's Gender** | **Infant's Weight (gr)** | **Gestation Weeks** |
| --- | --- | --- | --- |
| 1 | Female | 2630 | 38 |
| 2 | Female | 4400 | 39 |
| 3 | Male | 2780 | 39 |
| 4 | Female | 3300 | 40 |
| 5 | Male | 3900 | 40 |
| 6 | Male | 3120 | 40 |
| 7 | Female | 3410 | 39 |
| 8 | Male | 3251 | 40 |
| 9 | Female | 3150 | 40 |
| 10 | Female | 3010 | 38 |

**Figure S3.** Information regarding infant’s gender, weight and gestational weeks.


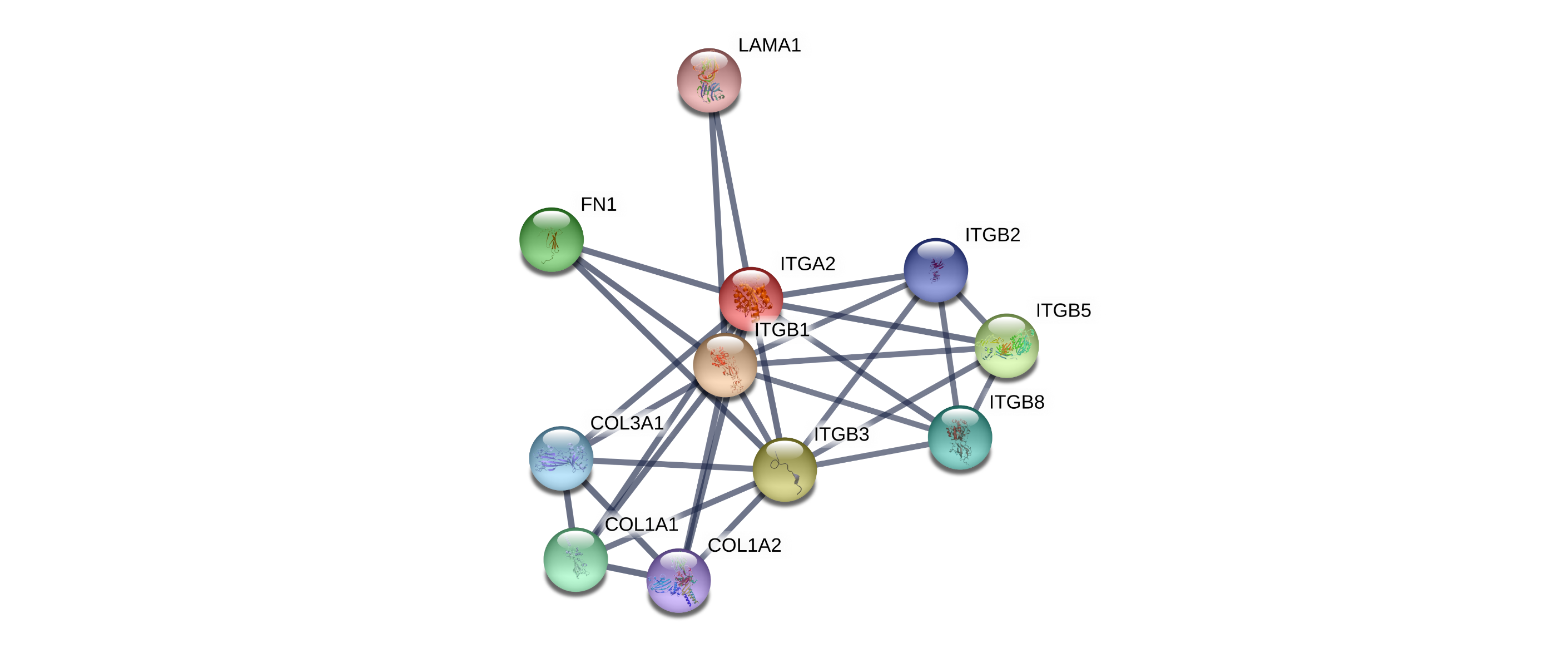
**Figure S4.** Interactions between ECM proteins of the decellularized hUAs with the adhesion molecules, based on analysis conducted in STRING database. Τhe initial input data in STRING database were COL3A1, COL1A2, FN1 and LAMA1, while all the other data represent possible interactions with those proteins. COL1A1: Collagen alpha-1 chain 1, COL1A2: Collagen 1 alpha-2 chain, COL3A1: Collagen alpha-3 chain, FN1: Fibronectin, LAMA1: Laminin, ITGA2: α2β2, ITGB1: α6β1, ITGB3: αIIβ3, ITGB5: αvβ5, ΙΤΓΒ2: αvβ2, ITGB8: αvβ8.
